# Bridging childhood to adulthood: the impact of early life stress on acute stress responses

**DOI:** 10.3389/fpsyt.2024.1391653

**Published:** 2024-04-18

**Authors:** Zheng Huang, Huizhi Bai, Ziyu Yang, Jingyu Zhang, Peishan Wang, Xiaoyu Wang, Liang Zhang

**Affiliations:** ^1^ Key Laboratory of Behavioral Science, Institute of Psychology, Chinese Academy of Sciences, Beijing, China; ^2^ Department of Psychology, University of Chinese Academy of Sciences, Beijing, China; ^3^ Key Laboratory of Modern Teaching Technology, Ministry of Education, Shaanxi Normal University, Xi’an, China

**Keywords:** early life stress, Trier social stress test, acute stress, stress recovery, heart rate

## Abstract

**Background:**

Childhood trauma exerts enduring impacts on the physical and psychological well-being of individuals in adulthood, influencing their daily functioning. This study aims to investigate the impact of childhood trauma on stress recovery in adults, concentrating on heart rate variations during acute stress exposure.

**Methods:**

A cohort of 126 participants completed the Childhood Trauma Questionnaire (CTQ) and underwent the Trier Social Stress Test (TSST) to elicit acute stress, with continuous heart rate (HR) monitoring for stress recovery assessment.

**Results:**

The results revealed a negative correlation between childhood trauma and stress recovery, prominently observed in instances of emotional neglect and abuse. Individuals with heightened childhood trauma exhibited protracted stress recovery following acute stress exposure.

**Conclusion:**

Childhood traumatic experiences were associated with the recovery from acute stress, as indicated by heart rate indices. These findings contribute to the foundational framework for psychological interventions tailored to individuals with a history of childhood trauma.

## 1 Introduction

Encountering a sudden and transient stressful event involves restoring homeostasis through the elicitation of nonspecific physiological and psychological responses, which is an essential adaptive ability honed through evolution (1). When confronted with a stressful event, individuals must respond promptly to optimize their adaptation (2, 3). These stress responses typically dissipate within a few minutes, returning to baseline physiological levels (4, 5), thereby restoring a normal physiological and psychological state. From a physiological perspective, an individual’s hypothalamic-pituitary-adrenal (HPA) axis and sympathetic nervous system (SNS) serve as the primary effectors in the stress response system (6, 7). The SNS functions by increasing blood pressure, skin electrical activity, and heart rate, thereby accelerating metabolism and providing energy to confront the stressor.

Stress adaptation varies significantly from one individual to another, influenced by the cumulative effects of innate factors and acquired long-term experiences. Childhood experience is a crucial determinant of individual differences in stress adaptation. Significant stress during childhood, such as abuse or ongoing or repeated physical or emotional trauma, can have lasting effects into adulthood. This sustained physical or psychological harm resulting from persistent abuse by parents or caregivers throughout childhood is referred to as childhood trauma (8–10). Numerous studies have established a link between childhood trauma and maladjustment in adulthood (11, 12). For example, individuals who have suffered childhood trauma are at a higher risk of developing cardiovascular disease, anxiety, and depression in adulthood compared to those without such a history (13–18).

One possible explanation for the aforementioned problems may be related to the fact that childhood trauma alters an individual’s response to and recovery from stress. As an early life stressor, childhood trauma is likely to have enduring effects on emotional regulation and coping mechanisms when confronted with subsequent stressors (19, 20). Research indicates that individuals with a history of childhood trauma tend to experience more pronounced negative effects from daily stressors compared to those without such a history (21, 22). Negative parenting behaviors during childhood, such as refusal or punishment, have been associated with lower resilience (23). Furthermore, individuals with a history of childhood trauma exhibit heightened psychological stress responses, report elevated stress levels, and consequently encounter increased psychological difficulties, thereby compromising their resilience (24, 25).

Moreover, previous research found that individuals with a history of adverse events in early life exhibit altered cortisol and heart rate responses to acute psychological stress (26–29). These alterations in physiological responses are attributed to early life stress. This change is a crucial factor in explaining why childhood trauma has long-term effects on individuals (18). As a significant physiological manifestation of stress, the response and recovery of the SNS (sympathetic nervous system) can exert a significant and long-term influence on an individual’s biopsychosocial health. Studies suggest that when these individuals encounter stress in adulthood, their childhood trauma experiences continue to affect their cardiovascular health, potentially leading to cardiovascular disorders characterized by either hyperreactive or blunted responses (30, 31).

Notably, most studies primarily concentrate on the physiological responses of individuals during stress, particularly the degree of SNS activation. However, it is crucial to consider not only how an individual reacts to stress (e.g., elevated heart rate) but also the efficiency of their recovery process. This is because if the individual fails to restore homeostasis after the stressor has subsided, the cumulative effects of stress can lead to deleterious consequences over time (32, 33). Consequently, Stress recovery, defined as the rapid adaptation and restoration of basic physiological levels after acute stress events, is a fundamental capability (34, 35). For instance, heart rate recovery measures, rather than reactivity measures, from cognitively challenging tasks could predict blood pressure changes in older healthy adults over a three-year period (36). Furthermore, another study demonstrated that better heart rate recovery from a mental arithmetic task is associated with a smaller carotid intima–media thickness (IMT), a marker of generalized atherosclerosis (37). Additionally, delayed or attenuated heart rate recovery after physical exercise predicts endothelium dysfunction (38) and coronary events (39).

Despite the significant health implications of heart rate recovery on coronary health and blood pressure, it has received limited attention in the context of physiological conditions related to childhood trauma. Only a handful of studies have employed heart rate recovery from stressful tasks as an indicator of coping. One study used a role-play task to investigate how psychosocial stressors impact blood pressure and heart rate among participants with varying family relationships. However, the study did not reveal any significant differences in heart rate recovery between the groups (27). Another study, utilizing the Cold Pressor task and the Stroop task as stressors, found that participants who met the criteria for childhood trauma took longer to recover their heart rate from the Cold Pressor task, but not the Stroop task (40). Therefore, for those who have experienced childhood trauma, attenuated heart rate recovery from more physical (such as the Cold Pressor task) has been confirmed, but it remains uncertain whether psychosocial stressor stressors can elicit a similar effect.

In summary, childhood trauma has a profound negative impact on daily life, affecting the physical and psychological well-being of individuals in adulthood and hindering their normal development. To address the question of the various enduring psychological and physiological risks associated with childhood trauma, it is crucial to understand how the physiological systems of traumatized individuals’ function when faced with stress. Previous studies have primarily focused on the effects of childhood trauma on cortisol and heart rate reactivity, but they have rarely explored its relationship with stress recovery. Therefore, this study employed the Trier Social Stress Test (TSST) to induce stress in individuals and aimed to investigate the impact of childhood traumatic experiences on stress recovery in adulthood. Our hypothesis was that childhood traumatic experiences could predict individuals’ stress recovery in adulthood. Specifically, we anticipated that individuals who experienced less childhood trauma would demonstrate higher levels of adaptation and recover more quickly from stressful situations.

## 2 Methods

### 2.1 Participants

Data were obtained from 126 students (female = 64; male = 62) aged 17 to 28 (M = 22.44, SD = 2.46). The study was voluntary, with participants informed of their confidentiality and the option to withdraw at any time. Therefore, everyone filled in a written informed consent form before the experiment and received appropriate compensation at the end. The Ethics Committee of Human Experimentation in the Institute of Psychology authorized the research proposal to guarantee that research ethics principles were followed. The experiment adhered to the following screening criteria to eliminate potential interfering factors: 1) excluding individuals who have been participated in stress-inducing experiments before; 2) excluding individuals with endocrine disorders, such as thyroid disease or adrenal disease, and those who had taken medications related to endocrine diseases, such as hormonal drugs, in the past month; 3) excluding subjects with psychiatric or neurological disorders; 4) excluding individuals with periodontitis or any wounds in the mouth, including oral ulcers; 5) excluding subjects who had colds, allergies, or acute exacerbation of chronic diseases in the past two weeks, and those who had taken any medications during this period; 6) excluding individuals who consumed liquor excessively (exceeding 100g per day) or smoked excessively (more than five cigarettes a day); 7) excluding subjects with long-term anxiety, depression, insomnia, or other similar symptoms, as well as those with irregular day and night work patterns; 8) ensuring that the body mass index (BMI) was within the normal range; 9) ensuring normal vision or corrected vision.

### 2.2 Procedures

**Figure 1:**
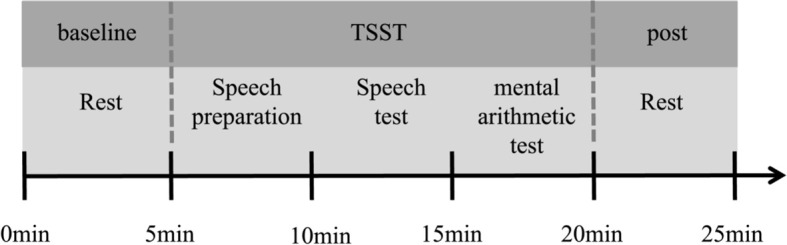
General procedure. Upon arrival at the laboratory, participants initially completed questionnaires and then rested for half an hour. Following this rest period, they measured their heart rate (HR) at rest for a 5-minute period to establish a baseline. Subsequently, they engaged in a stress task i.e., the Trier Social Stress Test (TSST). The TSST comprised three segments: a 5-minute preparation for a speech, a 5-minute public speech presentation, and a 5-minute public mental arithmetic task. During this process, participants wore a HR belt to monitor their HR. Following the TSST, participants returned to the preparation room to complete a 5-minute HR test in the resting state.

Upon arrival at the laboratory, subjects were first given a detailed explanation of the entire process and what would be covered in the experiment, they were also required to reconfirm that the screening criteria were met. It is important to note that on the day of the experiment, participants were not allowed to smoke, drink alcohol, strong tea or coffee, or engage in strenuous exercise within 2 hours before the experiment. After that, an informed consent form was signed. After a short break, they were instructed to complete a baseline mental arithmetic task and a series of questionnaires. The mental arithmetic task was carried out on the computer, which involved subtracting the number 14 consecutively from 1023 and entering the result. If any errors were made during the process, they were required to restart from 1023. Questionnaires included the demographic information questionnaire and the CTQ. Upon completion of the previous tasks, participants were given a 30-minute break. During this time, they were fitted with a heart rate monitor, and their baseline heart rate was recorded. Following the break, participants were provided with an additional 5-minute period of rest, during which they were instructed to remain seated and avoid any unnecessary movement. Afterward, they participated in the Trier Social Stress Test (TSST). After the TSST task, they return to a quiet room to rest. Heart rate data were continuously recorded throughout the entire experiment for subsequent analysis. The whole experimental procedure is shown in **Figure 1**.

### 2.3 Measures

#### 2.3.1 The Childhood Trauma Questionnaire

The Childhood Trauma Questionnaire (CTQ; 41, 42) is one of the world’s most widely recognized questionnaires for the assessment of childhood trauma experience. It has been revised and used by scholars from various countries and has good reliability and validity. This study used a Chinese version translated by Zhao et al. (43), which has been verified in the samples of middle school students and college students (44).

There are five subscales in CTQ, including emotional abuse (e.g., Someone in my family has said mean or insulting things to me.), physical abuse (e.g., Someone in my family beat me so badly that I had to go to the hospital.), sexual abuse (e. g., I think I was sexually abused.), emotional neglect (e. g., Home is the source of my strength and support.), and physical neglect (e. g., Someone looked after me and protected me.). It uses a five-point Likert scale (ranging from 1 = never to 5 = always), and each subscale contains five items, requiring participants to choose according to how often they experienced the situation before the age of 16. The higher the score, the more severe the participants’ childhood trauma. In this study, the sum of the items in the subscale was used as the subscale score, and the sum of the subscales was used as the total score of the CTQ (α = 0.71).

#### 2.3.2 Trier social stress test

In this study, the Trier social stress test (TSST) was used to trigger the participants’ stress response, which was developed by Kirschbaum et al. (4) and improved by Buchanan et al. (45). It is divided into three stages: speech preparation, speech test, and mental arithmetic test, each limited to five minutes.

Prior to the commencement of the experiment, participants were briefed in a preparation room about the task they would undertake. The scenario involved participants being informed that they were accused of theft by supermarket security guards while shopping. They were then instructed to prepare a speech in defense of their innocence, which they would later present to a panel of “supermarket managers”. During the speech preparation phase, participants were allowed to take notes, but they were expected to deliver their speech without referring to these notes.

After the preparation phase, participants were escorted to a separate room where they were required to perform the speech and mental arithmetic tasks. In this room, they encountered a panel composed of three “supermarket managers” – one male and two females – who were unfamiliar to the participants. The “managers” wore white coats and maintained a neutral and serious demeanor throughout the task to ensure the scenario’s credibility.

Immediately following the instruction to begin, participants commenced their timed five-minute speech, defending their innocence. Upon completion of the speech, participants were then instructed to perform a mental arithmetic task, which involved subtracting 13 consecutively from 1,022 and verbally reporting the result at each step. If a mistake was made, participants were required to restart the count from 1,022. The entire procedure, encompassing both the speech and mental arithmetic tasks, was recorded on video for subsequent analysis.

#### 2.3.3 Heart rate

In this study, we utilized the Polar H10 chest strap device and the Polar RS800CX watch to measure and record heart rate (HR), expressed as the number of heartbeats per minute (bpm). The Polar H10 sensor features a high data sampling rate of 1000 Hz and a sampling range of 30–240 bpm, allowing for precise measurement. It can be securely fastened to the chest area of each participant, and its validity for HR monitoring has been previously established (46). Additionally, the Polar RS800CX watch was used to synchronize with a data logger, ensuring the accurate recording and timing of HR data during the experiment. In addition to recording the HR of the whole experiment, this study also paid attention to the average value of several task stages. These stages include a 5-minute rest period, the TSST task period (a 5-minute speech preparation, a 5-minute speech test, a 5-minute mental arithmetic test), and a 5-minute stress recovery period.

### 2.4 Data analysis

In this study, statistical analysis was performed using SPSS version 23.0. First, descriptive statistics for various variables were computed. At the same time, we calculated the change value of HR as the stress recovery index. To observe the dynamic changes in stress recovery more clearly, this study quantifies it through the decline in heart rate during the first two minutes of acute stress events. Specifically, we used the HR of the first minute of the speech period minus the HR of the second minute of the speech period as the stress recovery value of the speech task (SR_HRsp = HRsp_1 − HRsp_2), and use the HR of the first minute of the mental arithmetic period minus the HR of the second minute of the mental arithmetic period as the stress recovery value of the mental arithmetic task (SR_HRco = HRco_1 − HRco_2). Analysis of variance (ANOVA) was then performed on the heart rate at different stages throughout the experiment to examine the effectiveness of stress induction. Secondly, a correlation analysis was conducted to explore the relationship between the scores of CTQ and its sub-scales with respect to the stress recovery indicators. Considering the potential impact of demographic factors, such as age, gender, and socioeconomic status, we calculated partial correlations between the CTQ scores, including its scaleless, and stress recovery while controlling for these demographic factors. Third, we examined the effect of CTQ on stress recovery through regression analysis. The CTQ scores that demonstrated a significant correlation with stress recovery in the aforementioned partial correlation analysis were employed as independent variables, while the stress recovery values were designated as the dependent variable.

## 3 Results

### 3.1 Descriptive statistics and preliminary analyses

The descriptive statistics results were shown in **Table 1**. The total score of CTQ (M = 32.61, SD = 5.81) ranged from 25 to 55. According to the average, we divided the participants whose total score was less than or equal to the median (score=31) into the low group, and the participants with the total score higher than the median into the high group. For overall, there were no significant differences in CTQ total score and sub scale scores between males and females. Similar results were found for the high and low groups. There was no significant difference in demographic variables (age, gender, and socioeconomic status) between the high and low trauma groups (*p*s > 0.05).

**Table 1:** The results of descriptive statistics and bivariate correlations (N = 126).

	M	SD	1	2	3	4	5	6	7	8	9	10	11
1. SR_HRsp	4.832	6.984	1										
2. SR_HRco	2.098	5.281	0.173	1									
3. Emotional abuse	5.960	1.597	−0.299**	0.034	1								
4. Physical abuse	5.357	1.236	0.034	−0.084	0.279**	1							
5. Sexual abuse	5.270	0.753	0.006	0.016	0.016	−0.027	1						
6. Emotional neglect	9.119	3.136	−0.268**	0.034	0.384**	0.204*	−0.044	1					
7. Physical neglect	6.905	2.207	0.021	0.009	0.208*	0.121	−0.004	0.435**	1				
8. CTQ (total score)	32.611	5.813	−0.211*	0.015	0.622**	0.442**	0.103	0.848**	0.697**	1			
9. gender	–	–	0.051	0.148	0.035	−0.153	0.015	0.022	−0.057	−0.030	1		
10. age	22.440	2.461	0.034	−0.054	−0.065	−0.149	−0.021	0.000	0.002	−0.051	−0.077	1	
11. Socioeconomic status (SES)	4.889	1.404	0.114	0.033	−0.113	−0.078	0.044	−0.104	−0.205*	−0.176*	0.081	−0.088	1

**p<.01,*p<.05.

**Figure 2:**
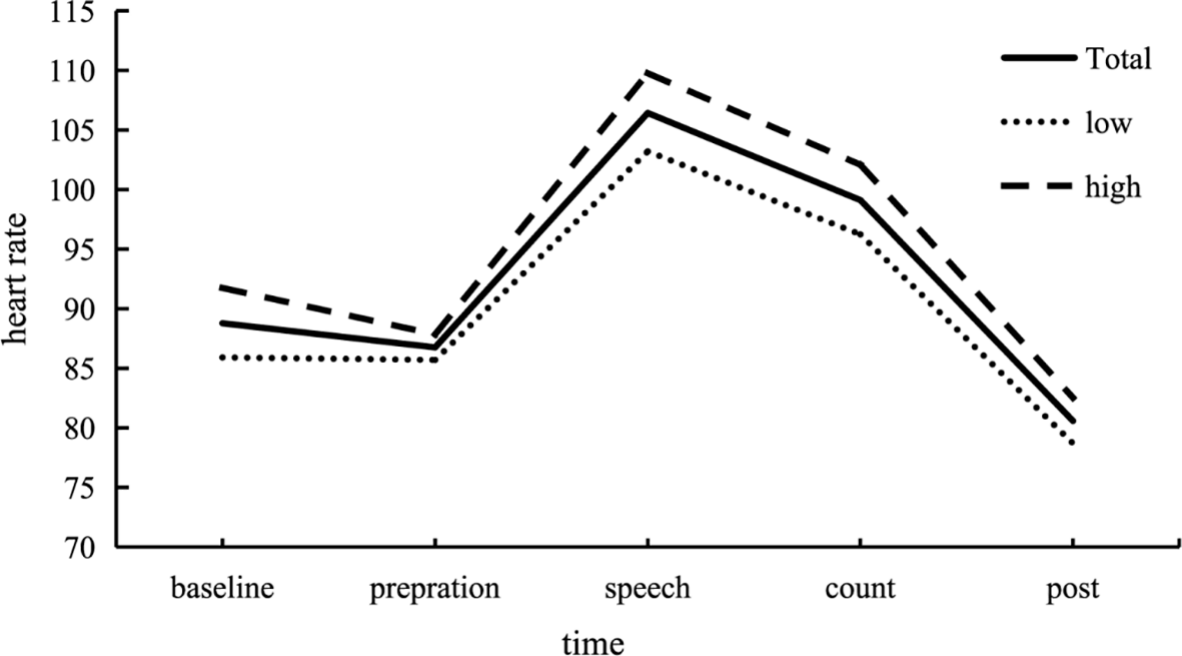
Changes in heart rate (HR) across each stage of the experiment. Participants exhibited increased stress levels during the Trier Social Stress Test (TSST). HR during the stress test, encompassing both the speech and mental arithmetic test periods, was significantly higher compared to the baseline, preparation, and post-test phases. This pattern was consistent across both the high and low trauma groups. In the figure, “Total” represents the response curve for all participants, “Low” represents the low trauma group, and “High” represents the high trauma group. In order to observe the dynamic changes of stress recovery more clearly, the change of heart rate per minute in the process of individuals facing stressful events was shown in **Figure 3**.

Further analysis of variance (ANOVA) was performed on the heart rate at different stages throughout the experiment, and the results were shown in **Figure 2**. For the whole group, participants experienced higher stress levels during the speech test and the mental arithmetic test periods. *Post hoc* tests revealed that heart rate during the TSST (the speech test and the mental arithmetic test periods) were significantly higher than all other periods (*ps* < .001). Similar results were found for the high and low groups.

**Figure 3:**
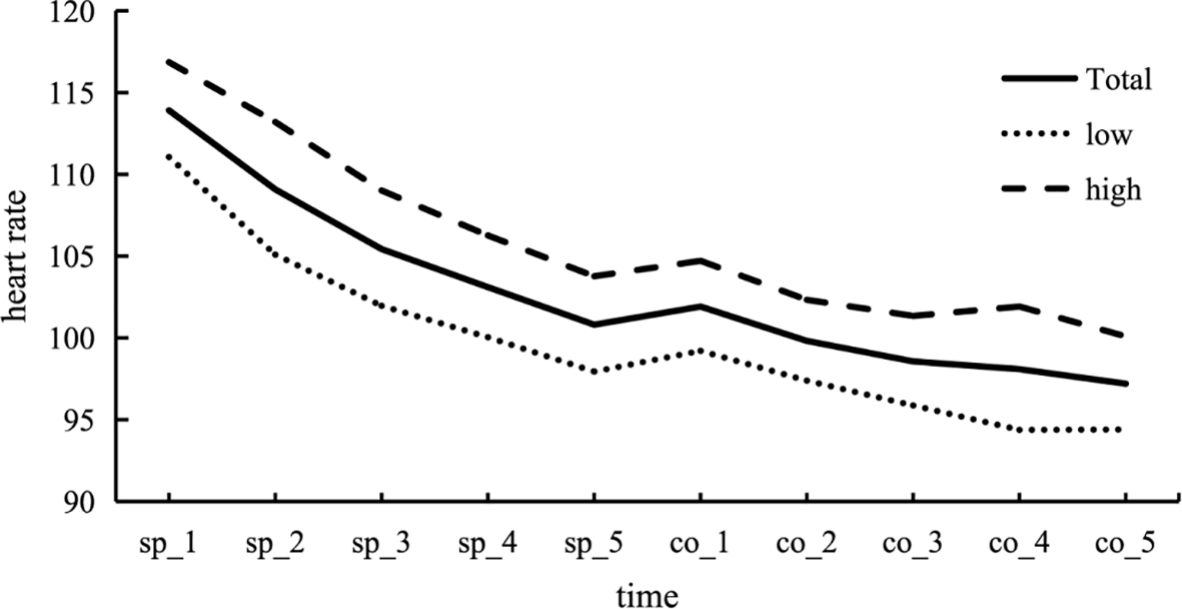
Changes in heart rate (HR) during acute stress events. During the speech period, HR tends to gradually decrease, indicating the occurrence of adaptive changes. Upon transitioning to the counting period, there is a slight upward trend in heart rate, which is followed by a decrease as the counting period continues.

### 3.2 Regression analysis

The results of the correlation analysis of the variables in this study were shown in **Table 1**. SR_HRsp were shown to be negatively associated with emotional abuse (*r* = −0.299, *p* = .001), emotional neglect (*r* = −0.268, *p* = .002) and the total score of CTQ (*r* = −0.211, *p* = .018). Physical neglect was found to be significantly negatively correlated with family social-economic status (*r* = −0.205, *p* = .021). And there was significant negative correlation between the total score of CTQ and family social-economic status (*r* = −0.176, *p* = .049). The results of partial correlations reveal that the total score of CTQ is significantly correlated with stress recovery (r = −0.192, p < .05). For subscales scores, only emotional neglect (r = −0.261, p < .01) and emotional abuse (r = −0.290, p < .01) are significantly correlated with stress recovery.

We subsequently conducted regression analyses to investigate the impact of CTQ on stress recovery. Model 1 revealed that the overall CTQ score significantly predicted the reduction in heart rate (SR_HRsp) following the TSST (β = −0.211, *t* = −2.403, *p* = .018). To elucidate the contributions of specific subdimensions to stress recovery, we included the sub-scale scores that were significantly associated with SR_HRsp in the regression model. Model 2 indicated that emotional abuse (β = −0.230, *t* = −2.509, *p* = .013) and emotional neglect (β = −0.180, *t* = −1.960, *p* = .052) emerged as significant predictors of SR_HRsp in response to the TSST.

To account for potential demographic influences, we constructed two additional regression models that controlled for age, gender, and family socioeconomic status, as detailed in **Table 2**. The findings remained robust after adjusting for these demographic variables. The total CTQ score continued to significantly predict SR_HRsp to the TSST (β = −0.194, *t* = −2.152, *p* = .033). Among the sub-scales, emotional abuse remained a significant predictor of SR_HRsp (β = −0.224, *t* = −2.407, *p* = .018), while emotional neglect approached significance (β = −0.176, *t* = −1.904, *p* = .059). Notably, higher levels of emotional abuse and emotional neglect were associated with poorer SR_HRsp.

**Table 2:** Results of multiple regression analyses.

		SR_HRsp		
		*β*	SE	*t*
Model 1	Age	0.035	0.253	0.388
	Gender	0.041	1.238	0.460
	Socioeconomic status (SES)	0.080	0.450	0.883
	CTQ(total score)	−0.194	0.108	−2.152*
	*R* ^2^	0.053		
	*F*	1.703		
Model 2	Age	0.031	0.244	0.354
	Gender	0.059	1.195	0.690
	Socioeconomic status (SES)	0.069	0.431	0.791
	Emotional abuse	−0.224	0.407	−2.407*
	Emotional neglect	−0.176	0.206	−1.904
	*R* ^2^	0.126		
	*F*	3.468**		

**p<.01, *p<.05. CTQ – Childhood Trauma Questionnaire; SR_HRsp – the heart rate (HR) recovery value in the speech task. Model 1 demonstrated that the total score on the CTQ significantly predicted the decrease in stress recovery (SR_HRsp) subsequent to the Trier Social Stress Test (TSST). Model 2 showed that emotional abuse and emotional neglect were significant predictors of SR_HRsp in response to the TSST, with emotional neglect approaching significance (p = 0.059). To account for potential demographic influences, age, gender, and family socioeconomic status were included as control variables.

## 4 Discussion

The objective of this study was to investigate the impact of childhood trauma experiences on stress recovery during laboratory-induced acute stress. The findings revealed that when individuals encountered acute stress situations, their heart rate initially spiked and then gradually decreased. There was a negative correlation between stress recovery and the scores of CTQ. Specifically, individuals who had experienced more severe childhood trauma exhibited a slower return of their heart rate to baseline levels after exposure to the stressful event. This finding was further supported by regression analysis.

Childhood trauma negatively predicted an individual’s stress recovery, confirming the hypothesis of this study. However, this finding contradicts some previous results. In one study, researchers did not observe any significant difference in heart rate recovery between participants with positive and negative family relationships after a stressful role-play task (27). Participants were asked to role-play a challenging social situation, where they had to ask a neighbor to turn down loud music, but the neighbor refused. Although their heart rate increased significantly during the task, but even in the negative family relationship group with a higher heart rate, their average heart rate remained below 96 beats per minute (bpm). In contrast, during the speech period of the Trier social stress task in our study, our participants had an average heart rate of over 106 bpm which is higher than their results. It is possible that the Trier social stress task elicited a stronger heart rate response, enabling it to detect heart rate recovery with more precision.

In another study, researchers found significant differences in heart rate recovery after the Cold Pressor task between a childhood trauma group and a non-childhood trauma group, but this effect was not observed after the Stroop task. Unfortunately, they did not provide specific heart rate data for both tasks, rather using “proportion unrecovered” as the index of heart rate recovery. However, according to one study investigating the cardiovascular response to the Stroop task, the average heart rate during different variations of the Stroop tasks remained below 75 bpm (47), so it’s still possible that the Stroop task does not elicit enough heart rate response to detect differences during the recovery period. The heart rate recovery pattern observed in different groups during the Cold Pressor task aligns with our finding, potentially validating our hypothesis. This might also indicate that heart rate response and recovery do not differentiate between a more physical stressor (cold exposure) and a more psychosocial one (public speech). Taken together, several studies have yielded similar results to ours, but it still needs further investigation whether the lack of sufficient heart rate response is the reason why the aforementioned studies did not find any differences between childhood trauma/non-childhood trauma group, and if this is the case, how much cardiovascular response criteria it has to meet to detect such differences.

Previous studies examining the response and recovery of blood pressure following laboratory-induced acute stress can further support the current hypothesis. After investigating a large sample of middle-aged and elderly individuals, some researchers have observed that individuals who experienced high-intensity psychosocial stress during childhood tend to exhibit slower recovery of blood pressure from acute stressors (48). Additionally, other studies have indicated that delayed recovery of cardiovascular reactions, such as elevated blood pressure and heart rate, may be indicative of stress response disorder (48, 49).

Another focal point of this experiment was to examine which aspects of childhood trauma contribute to reduced stress recovery following psychosocial stressors. We will delve deeper into this discussion in the following sections.

Childhood is a particularly sensitive and critical stage in brain development, where biological processes related to long-term stress responses can undergo significant changes. Furthermore, the effects of environmental exposure during this period are often more pronounced than in adulthood (50). On the one hand, traumatic events in childhood will have a negative impact on the development of the stress response system (25, 51). Childhood trauma may impair the normal activity of the HPA axis function (52), decreased function of the locus coeruleus-norepinephrine (LCNE) system (53, 54), and affect the production of oxytocin (55), all of which are associated with stress disorder. On the other hand, adversity in early childhood may be associated with changes in brain structure and functions of the brain systems responsible for regulating emotion and motivation (56–58). In essence, childhood trauma can impact an individual’s ability to recover from acute stress more slowly, mediated by the neural mechanisms underlying emotional and motivational processes.

More specifically, emotional abuse and emotional neglect in childhood trauma can negatively predict stress recovery in adulthood, thereby confirming our research hypothesis. Our findings established a direct correlation between exposure to emotional abuse or emotional neglect and the ability to recover from stress. Previous studies suggested that the type of emotional support received in childhood has a profound impact on adult life (59–63). One of the reasons emotional supports is so influential in stress recovery during adulthood is its association with resilience. Resilience is the ability to adaptively overcome stress and adversity while maintaining normal psychological and physiological functions, facilitating rapid recovery after adverse experiences (64, 65). A study investigating the relationship between recalled parental rearing behavior and resilience found that participants who experienced positive rearing behavior during childhood exhibited higher resilience, while those who experienced negative rearing behavior, such as rejection and punishment exhibited lower resilience (23). Furthermore, studies have linked emotional neglect with lower self-efficacy (16), which is considered a crucial component of resilience (66).

Thus, individuals who have experienced severe childhood trauma tend to exhibit lower psychological resilience in the absence of intervention by other external factors. Consequently, when confronted with acute stress events in adulthood, they often lack sufficient psychological resources to deal with stress, resulting in a prolonged recovery process. Notably, the recovery of their physiological state closely mirrors their psychological resilience (25). Conversely, individuals who have experienced less childhood trauma are more likely to recover swiftly from acute stress in adulthood. Individuals with high self-esteem and strong social support (including family and friends) are better equipped to mitigate anxiety and perceived stress following acute stress events (25, 34, 67). Importantly, individuals with good resilience not only recover more quickly from psychological difficulties following significant stress or trauma but also have a lower risk of developing psychological disorders (25).

There is no doubt that childhood trauma as a whole could affect mental wellbeing, but some researchers hypothesize that various forms of childhood trauma can differently affect stress response in adulthood. Researchers categorized childhood trauma into two broad categories: deprivation and threat. Deprivation encompasses a wide variety of topics, including emotional neglect, physical neglect, poverty, and institutionalization. On the other hand, threats may include emotional and physical abuse, emotional and physical bullying by peers, sexual abuse, and exposure to community violence. It is hypothesized that exposure to deprivation in childhood may lead to a reduction in the thickness and volume of the association cortex, while exposure to threats during childhood can affect the hippocampus, amygdala, and ventromedial prefrontal cortex, ultimately influencing fear conditioning and extinction (68).

There have been several studies of the HPA-axis confirming this categorization. One study specifically examined the impact of different types of childhood adversity on cortisol indicators among teenage participants aged 9–16. These indicators include cortisol awakening response, diurnal cortisol regulation, acute stress reactivity, and recovery. They found out that exposure to physical abuse accelerates HPA-axis activation to a stress task (Socially-Evaluated Cold Pressor Task) and exposure to emotional abuse hinders cortisol recovery following the task (69). A second study distinguished between deprivation (operationalized as poverty) and threat (operationalized as exposure to violence) in terms of psychopathology and physiological reactivity among teenagers. They found out that threat but not deprivation is associated with differences in the HPA axis indicator (70). A third study found that exposure to childhood adversity is related to elevated morning cortisol levels, but this association is confined to threat exposure in females (71). Finally, a meta-analysis study confirmed that exposure to uncontrollable threats is associated with a high, flat diurnal profile of cortisol secretion (72).

In these aforementioned studies, only exposure to threat or abuse could elicit anomalies in the HPA-axis, but how different types of childhood adversity affect the autonomic system is less documented, and existing studies exhibit some inconsistency regarding whether threat and deprivation will affect the autonomic nervous system (ANS) to the same degree. One study, for instance, indicated that deprivation does not influence the ANS as profoundly as threat does. In Busso et al.’s study (70), along with measuring HPA axis indicators, they also assessed ANS markers such as respiratory sinus arrhythmia (RSA) and pre-ejection period (PEP). The hierarchical linear regression model revealed that after accounting for interpersonal violence, deprivation (poverty) alone did not significantly alter the pre-ejection period (PEP) during the TSST test (70). Nevertheless, some studies suggest early-life deprivation can indeed affect the ANS. One such study compared three groups of children: institutionalized children who later received high-quality foster care, institutionalized children who remained in standard care, and children who were never institutionalized. The researchers found that, compared to the children who received high-quality foster care, the institutionalized children who stayed in standard care exhibited a notably blunted ANS response, as indicated by heart rate, diastolic blood pressure (DBP), and pre-ejection period (PEP) during the speech component of the TSST test, as well as pre-ejection period (PEP) during the math component (73). Another study also focused on institutionalized children, comparing the autonomic response of late-adopted children (adopted after 12 months), early-adopted children (adopted before 8 months), and non-adopted children (raised by their birth parents) during the TSST-C test. The findings revealed that late-adopted children exhibited a lower overall PEP compared to non-adopted children (74). Notably, both studies lacked a comparison group exposed to threat, limiting the ability to draw conclusions about the relative impact of deprivation and threat on the autonomic system.

In our study, we encompassed both neglect (emotional neglect, physical neglect) and threat (physical abuse, emotional abuse, and sexual abuse) exposure, and instead of focusing on the reactivity of the ANS, we delved into the recovery process. Our results suggested that both emotional abuse and emotional neglect led to delayed heart rate recovery following the TSST experiment. This may suggest that various forms of childhood trauma impact the heart rate recovery process similarly, yet this conclusion warrants further investigation. Given the frequent coexistence of abuse and neglect, a more meticulous experimental control is imperative.

Several limitations need to be mentioned in the present study. First of all, this study employed a cross-sectional design, which provided a snapshot of the relationship between childhood trauma and stress recovery in adulthood. However it does not permit the establishment of causality. The temporal sequence of events, which is essential for inferring causal relationships, cannot be accurately determined from cross-sectional data. This limitation suggests that future research should employ longitudinal designs that can track the progression of stress responses and recovery over time, thereby offering a clearer understanding of how childhood experiences may influence later physiological and psychological outcomes. Second, this study used the self-reported CTQ to assess childhood abuse, and most of the individuals’ scores were concentrated in the mild to moderate range, which resulted in an uneven distribution of various types. Future studies will screen according to the severity and expand the sample size to further test the recovery from acute stress in individuals with different types of CTQ. Third, the sample in this study consisting of individuals from a similar age range, poses limitations to the generalizability of our results. Future research could expand the age range to include both younger and older individuals, as well as recruiting participants from various occupational and socioeconomic backgrounds. Last, this study only used TSST to induce acute stress states in individuals but did not clearly distinguish the type or severity of stressors. Additionally, our study did not examine psychological resilience or individuals’ interpretation of past experiences, which may be crucial factors affecting stress recovery in adulthood. Future research should explore the potential mediating role of resilience in the relationship between childhood trauma and adult stress responses. Incorporating such measures could offer a more detailed understanding of the variables that underlie the variability in stress recovery among individuals.

In summary, this study explored the effects of different childhood traumatic experiences on stress recovery under laboratory-induced acute stress. The results found that when individuals faced acute stress, the more severe the trauma experienced in childhood (especially emotional neglect and emotional abuse), the slower the stress recovery. This also provides a further theoretical basis for psychological intervention for individuals with childhood trauma. To reduce the impact of childhood trauma on individuals, teachers or social workers can provide psychological resilience intervention courses and call on people around them to provide strong social support.

## Data availability statement

The raw data supporting the conclusions of this article will be made available by the authors, without undue reservation.

## Ethics statement

The studies involving humans were approved by The Ethics Committee of Human Experimentation in the Institute of Psychology, Chinese Academy of Sciences. The studies were conducted in accordance with the local legislation and institutional requirements. The participants provided their written informed consent to participate in this study.

## Author contributions

LZ: Conceptualization, Funding acquisition, Project administration, Supervision, Writing – review & editing. ZH: Conceptualization, Writing – original draft. HB: Formal analysis, Writing – review & editing. ZY: Formal analysis, Writing – review & editing. JZ: Funding acquisition, Writing – review & editing. PW: Data curation, Writing – review & editing. XW: Data curation, Writing – review & editing.
